# Nanonets Derived from Turnip Mosaic Virus as Scaffolds for Increased Enzymatic Activity of Immobilized *Candida antarctica* Lipase B

**DOI:** 10.3389/fpls.2016.00464

**Published:** 2016-04-11

**Authors:** Sol Cuenca, Carmen Mansilla, Marta Aguado, Carmen Yuste-Calvo, Flora Sánchez, Jose M. Sánchez-Montero, Fernando Ponz

**Affiliations:** ^1^Centro de Biotecnología y Genómica de Plantas, Universidad Politécnica de Madrid – Instituto Nacional de Investigación y Tecnología Agraria y AlimentariaMadrid, Spain; ^2^Grupo de Biotransformaciones, Departamento de Química Orgánica y Farmacéutica, Facultad de Farmacia, Universidad Complutense de MadridMadrid, Spain

**Keywords:** nanobiocatalysis, nanonets, turnip mosaic virus, chemical conjugation, enzyme nanoimmobilization

## Abstract

Elongated flexuous plant viral nanoparticles (VNPs) represent an interesting platform for developing different applications in nanobiotechnology. In the case of potyviruses, the virion external surface is made up of helically arrayed domains of the viral structural coat protein (CP), repeated over 2000 times, in which the N- and C-terminal domains of each CP are projected toward the exterior of the external virion surface. These characteristics provide a chemical environment rich in functional groups susceptible to chemical conjugations. We have conjugated *Candida antarctica* lipase B (CALB) onto amino groups of the external surface of the potyvirus turnip mosaic virus (TuMV) using glutaraldehyde as a conjugating agent. Using this approach, TuMV virions were transformed into scaffolds for CALB nanoimmobilization. Analysis of the resulting structures revealed the formation of TuMV nanonets onto which large CALB aggregates were deposited. The functional enzymatic characterization of the CALB-bearing TuMV nanonets showed that CALB continued to be active in the nanoimmobilized form, even gaining an increased relative specific activity, as compared to the non-immobilized form. These novel virus-based nanostructures may provide a useful new approach to enzyme nanoimmobilization susceptible to be industrially exploited.

## Introduction

Enzyme immobilization is a common practice widely adopted in many industrial biochemical processes (DiCosimo et al., 2013; Sheldon and van Pelt, 2013). The main objective of enzyme immobilization is to enable the creation of biocatalysts able to mediate the synthesis of desired products with an increased suitability for industrial needs, such as homogeneous catalytic activity, catalyst reutilization, and/or the ability to separate substrates and products in different media. Immobilized enzymes can provide additional advantages like an increased resistance to potential factors that stress enzymatic activity, for instance pH, pressure, temperature or solute concentrations (Mateo et al., 2007; Branco et al., 2015). Notwithstanding the clear advantages, immobilization processes often take place with an associated loss of enzyme specific activity (Sheldon and van Pelt, 2013). This undesirable side effect may result from protein rigidity or lack of mobility imposed by enzyme immobilization. Frequently, however, the overall advantages are superior to the disadvantages, and hence, use of immobilized enzymes continues to increase.

The sophistication and complexity of immobilization technology has grown over time, along with the development of many variants. One of the most rapidly adopted of these variants involves enzyme immobilization at the micro- and nanoscales (Cipolatti et al., 2014). Several arguments have been raised that favor the implementation of nanoscale immobilization. For example, an increased stability in comparison with the macroscale; toxin-free and relatively simple fabrication processes; homogeneity in nanoparticle covering; relative surface and enzyme load increments; and an important reduction of the limitation imposed by mass transfer, are notable pros favoring operations at the nanoscale level (Cipolatti et al., 2014). Most advances so far have occurred with the deployment of inorganic nanoparticles duly functionalized chemically in order to retain enzymes within them or at their external surfaces. A large range of inorganic nanoparticles have been exploited for this purpose. Among them carbon nanotubes, varying types of magnetic nanoparticles, and gold or metallic oxides nanoparticles are often cited in the literature, alone or in combination (Sheldon and van Pelt, 2013). There are, however, important reasons to develop enzyme nanoimmobilization on organic proteinaceous materials, in addition to inorganic ones (Guauque Torres et al., 2014). Supporting proteins and enzymes have the same chemical composition (linear amino acid polymers) thus providing a desirable chemical homogeneity to the complexes. Protein-based nanocomplexes offer improved biocompatibility, an important advantage in human health and food-related processes. From a chemical standpoint, supporting proteins provide a wealth of chemical functional groups from the different amino acids thus opening a wide range of possibilities for enzyme immobilization through different conjugation approaches. A particular case of proteinaceous enzyme nanoimmobilization is self-conjugation. This approach gives rise to the so-called CLEAs (cross-linked enzyme aggregates), complexes increasingly used in recent years (Sheldon, 2011).

An interesting form of enzyme nanoimmobilization on proteinaceous supports combines this approach with the more canonical one of immobilizing enzymes on pre-existing nanoparticles. The main form of these nanoparticles can be found in virions and virus-like particles (VLPs), the ensemble frequently referred to as viral nanoparticles (VNPs) (Steinmetz and Manchester, 2011). VNPs are proteinaceous nanoparticles, that may be combined with nucleic acids and/or lipids, and having many of the advantages of both conventional inorganic nanoparticles and those of simple proteinaceous supports. The result is a situation that provides multiple possibilities for nanobiotechnological developments. A good number of these developments already can be found in the literature (Manchester and Steinmetz, 2009; Steinmetz and Manchester, 2011). VNPs of different geometries have been used for this purpose, mostly icosahedrons and rods.

In all cases VNPs need to be functionalized in some of their reactive chemical groups, in order to be able to conjugate with the enzyme to be immobilized. There are a number of possibilities to achieve this, and the issue has been extensively reviewed (Cardinale et al., 2012). Plant-derived VNPs present themselves as a significant source of such nanoparticles. Biosafety is of course an important advantage of plant-derived VNPs, since no plant virus has been reported as an infectious agent for humans or higher animals. Cost and efficiency of production are also relevant aspects to be considered (Vezina et al., 2011).

We have approached enzyme immobilization on the external surface of a plant virus, turnip mosaic virus (TuMV), an elongated flexuous virus belonging to the *Potyvirus* genus of the *Potyviridae* family (ICTVdB Management, 2006). We think that the geometry of TuMV VNPs presents important advantages for enzyme nanoimmobilization. Being elongated and flexuous, it is conceivable that they can derive supporting structures with a high potential for adaptation to different enzymes, since they should be able to bend, stretch, or even conjugate to themselves. We have tested these notions by immobilizing Lipase B from *Candida antarctica* (CALB) on TuMV using glutaraldehyde (GA) as a two-headed conjugating agent. The result was the formation of TuMV-based nanonets onto which large CALB aggregates formed. These supramacromolecular complexes proved to be active, even showing an increased enzymatic specific activity.

## Materials and Methods

### Virus Purification

Indian mustard (*Brassica juncea* L.) plants at developmental stage 1.4–1.5 (4–5 true leaves stage) were inoculated with TuMV UK1 isolate (Tomlinson and Ward, 1978) in order to propagate the virus. Purification of viral particles was done according to the protocol described (Sánchez et al., 1998). Purified virions were stored at -20°C in 0.25 M potassium phosphate buffer pH 7.5, 10 mM EDTA, 50% (v/v) glycerol. For virus quantification, Nanodrop^®^ measures were taken and the typical potyvirus extinction coefficient of 2.65 (A 0.1%, 1 cm at 260 nm) was used in the calculations.

### Virus Stability Assays

Virus stability assays were run in order to evaluate structural maintenance under different conditions, such as pH, pH of conjugation buffer, and organic solvents used in the activity assays. Structural distortions were assessed based on changes in the absorbance spectra and on the A_250_/A_267_ ratio typical of non-enveloped, elongated plant viruses with a helical symmetry (Thouvenel et al., 1976). Spectra were obtained in a NanoDrop^®^ device after 24 h at room temperature (RT). Stability determinations were calculated considering non-treated virus in final concentrations of 500 and 125 μg/mL as references. Samples included three technical and biological replicates (three samples, three measurements per sample).

pH-dependent stability was evaluated by adding the Britton and Robinson buffer (Britton and Robinson, 1931) in a pH range 5.0–12.0. Stability in the conjugation buffer (0.02 M phosphate buffer) was also assayed at pH 7.0, 8.0, and 9.0.

Since complexes developed in this paper will eventually be tried in organic media, structural preservation was also investigated, using acetonitrile as a model. Assays were run at the concentrations used in activity assays, 7 and 50% (v/v). Finally, viral stability at different temperatures was assessed by combining different times (60–360 min) and temperatures (50, 60, 70, and 100°C).

### Chemical Conjugation

**FIGURE 1:**
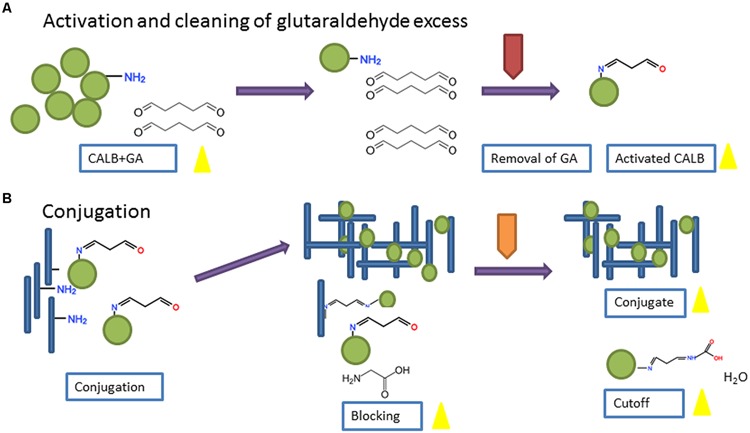
**Scheme of the procedure followed for bioconjugation of CALB and TuMV.** Parts **(A)** and **(B)** in the figure refer to the Activation and Conjugation processes respectively.

Bioconjugation was carried out using an adaptation of a published procedure (Britton and Robinson, 1931), schematically shown in **Figure 1**. *Candida antarctica* lipase b (CALB) from Sigma^®^ (catalog number 62288; 7.2–10.8 U/mg) was dissolved in 0.02 M phosphate buffer, pH 8.0, at a concentration of 1 mg/mL or 10 mg/mL and incubated with GA 1% (v/v) for 1 h RT under mild shaking. The excess of crosslinking agent was removed using commercial devices; a desalting column of acrylic resin with a molecular weight cutoff (MWCO) of 7 kDa (Zeba Spin column^®^) for the low concentration enzyme solution (1 mg/mL) and a membrane of regenerated cellulose with a nominal molecular weight limit (NMWL) 10 kDa (Amicon^®^ YM-10) for the more concentrated one (10 mg/mL). The recovered concentrate of CALB + GA was adjusted to the initial volume with phosphate buffer.

CALB + GA was virus-conjugated at different CP-TuMV:CALB ratios in a final volume of 600 μl. Ratios assayed were 100:1, 10:1 and 1:1 (units of CP:units of CALB). In this case ‘units’ refer to the number of protein molecules (CP or CALB) in the conjugation reaction. To estimate the number of protein units, we considered that both proteins have the same approximate Mw (33 kDa) and that the number of CP units in a TuMV virion is approximately 2000. Controls excluding GA or virus were included. Mixtures were incubated for 18 h at 4°C with orbital shaking (15 rpm). Remaining reactive groups after conjugation were blocked by addition of 0.5 M glycine (30 μl) in carbonate buffer, pH 8.0, followed by incubation 2 h at RT with mild shaking.

Mixtures were filtered in a commercial device with a NMWL of 100 kDa, (Amicon^®^ YM-100), according to the manufacturer protocols. Final volumes were set in order to achieve a desired final concentration with conjugation buffer. The resulting conjugates were stored at -20°C in 50% glycerol (v/v).

### SDS-PAGE

In order to estimate the amount of CALB linked to the virus, an SDS-PAGE (12%) was run and stained with EZ blue (Sigma^®^). Free lipase quantities ranging from 20 to 200 ng were loaded in parallel with different volumes of cutoff (obtained from exclusion volumes of Centricon^®^). CALB concentration of the cutoffs were determined by comparison of band intensities with Adobe Photoshop^®^ software. A 5% loss was considered in all samples due to extra bands not corresponding to the 33 kDa (CALB monomer) or 66 kDa (CALB dimer) bands. Both the 33 and 66 kDa bands were considered for quantification taking into account the initial amount available for conjugation and the amount detected on the exclusion volumes.

### CALB Activity Assays

Hydrolytic activity and kinetic assays were done with *p*-nitrophenyl acetate (pNPA) as a substrate as described (Jung and Park, 2008), using 50 mM MOPS-Na, pH 8.0. Activity assays were developed in 96-well microplates, 30 min at RT with shaking, in a final volume of 250 μl. Absorbance was measured with a Tecan^®^ spectrophotometer at 405 nm. Comparisons among different steps of the process were done at 1.5 mM substrate concentration. Duplicates and a blank control were included. Activity was calculated using different amounts of each mix in the steps. The Michaelis–Menten constant (Km) and the maximum velocity for the reaction (Vmax) with pNPA as substrate, were calculated by fitting the kinetic results to a Michaelis–Menten curve (shown in **Supplementary Figure S5**) using Origin v8.0 software. Fold-increases were referenced to commercial CALB by slope comparison.

### Electron Microscopy

Electron microscopy samples were prepared laying a 10 μl drop of the conjugates and controls on Formvar^®^ nickel-coated electron microscopy grids (3.05 mm nickel grid, TAAB^®^). After 15 min of incubation, grids were washed with 200 water droplets and stained with 10 μl 2% (w/v) uranyl acetate during 2 min. Grids were dried and stored at RT. Grids were inspected by transmission electron microscopy (JEM JEOL1010) at 80 Kv.

## Results

### Virus Purification

The virus purification process yielded approximately 30 μg of virus per gram of infected tissue. The coat protein (CP) size was ca. 33 kDa, according to its SDS-PAGE mobility (data not shown).

### Virus Stability

Virion stability with respect to several external conditions is a critical subject for further conjugation approaches. Thus, we analyzed their UV optical properties under different conditions of pH, temperature, solvents (acetonitrile), and conjugation buffer. An unchanged A_250_/A_267_ ratio under the conditions tested was taken as evidence of an unaltered structure.

**FIGURE 2:**
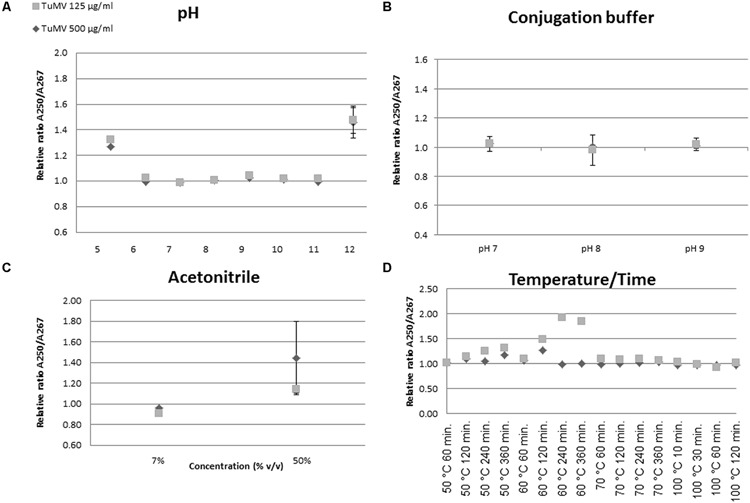
**UV spectroscopy assessment of structural changes in virion structure.** Structural changes were assessed by calculating the A_250_/A_267_ ratio. Unaltered virions show a ratio of 1.00. The different panels **(A–D)** show representations of changes in this ratio of virions subjected to the different conditions.

The A_250_/A_267_ ratio between samples and controls did not change in the pH range 6.0–11.0 of the Britton and Robinson buffer, indicating no detectable alterations of the viral particles in this range (**Figure 2A**). An increase in this ratio was found at pH 5.0 and pH 12.0, indicating some relevant change in virion structure. The results showed no variation at different virus concentrations (125 and 500 μg/mL). The ratio increase observed at pH 5.0 and pH 12.0 was also evident when whole UV spectra were obtained at varying pH values (**Supplementary Figure S1**). These results are consistent with the assertion that chemical conjugations performed in the 6.0–11.0 pH range do not affect overall virion structure. For further conjugation assays the phosphate buffer was tested at three different pHs (7.0, 8.0, and 9.0). Using similar measurement techniques, it appears that the structure remained largely unchanged, although small variations in the A_250_/A_267_ ratio were observed at pH 8.0 – the pH chosen for conjugation assays, according also to previous work by other authors (Wine et al., 2007) (**Figure 2B**).

Maintenance of structure in acetonitrile was also tested at different concentrations. The relative A_250_/A_267_ ratio remained close to 1 at 7% (v/v), the concentration used in the activity assays (**Figure 2C**). Changes in this ratio were found at higher percentages (i.e., 50% v/v). In addition, temperature stability was assayed. The expected absorbance ratio was maintained at 50°C over 240 min, after which the structure changed, likely to a non-stable transitional state. At higher temperatures, such as 60°C, the A_250_/A_267_ ratio increased even after short times. Somewhat surprisingly, at 70 and 100°C little variation between samples was detected (**Figure 2D**).

### Chemical Conjugation

Conjugation was performed as described in the “Materials and Methods.” Five reactions were carried out in parallel with the conjugates shown in **Table 1** (C1–C5). The chemical reaction model for interaction among CALB proteins and virions is via amino groups (**Supplementary Figure S2A**). This agrees with the color variation observed in conjugates after the blocking step, becoming increasingly dark from low to high ratios of TuMV:CALB, an effect most likely due to the formation of Schiff bases (**Supplementary Figure S2B**).

**Table 1:** Characteristics of conjugates and names.

Characteristics	Sample name
TuMV-CP:CALB 1:0.01	C1
TuMV-CP:CALB 0:1	C2
TuMV-CP:CALB 1:0.01 (without GA)	C3
TuMV-CP:CALB 1:0.1	C4
TuMV-CP:CALB 1:1	C5

After blocking, conjugates were filtered through a device with a 100 kDa exclusion limit, thus CALB linked to or trapped by TuMV particles should be retained, together with supramacromolecular complexes formed by at least four CALB units. To evaluate the proportion of CALB retained by the separation devices, quantification of protein in the exclusion volumes was done, based on band intensity after electrophoretic separation. Loading known concentrations of free lipase a calibration curve was obtained with an *R*^2^ = 0.97. An average background value was used to quantify bands, considering both the 33 and 66 kDa bands (**Supplementary Figure S3**). Image analysis software was applied, CALB linked to the virus was estimated, and the percentage of virus-linked lipase was determined (**Supplementary Figure S4**). The highest percentages were achieved by C5, although C4 results were similar (**Table 2**).

**Table 2:** Estimated efficiency of conjugation presented as CALB percentage contained in each final sample.

Sample	Estimated ng/μl in PAGE	Estimated ng in cutoff	CALB initial ng	ng CALB linked or entrapped	% CALB linked or entrapped
C1	2.4	1783.8	2400	616.2	25.7
C2	2.1	1640.4	2400	759.6	31.7
C3	1.1	401.4	1200	798.6	66.5
C4	1.8	1370.0	24000	22630.0	94.3
C5	19.9	13873.6	240000	226126.4	94.2

### CALB Activity Assays

Kinetic assays were carried out using variable substrate concentrations. Differences in activity were obtained at different points of the immobilization protocol (not shown). Zeba columns^®^ provided a notable improvement of activity for samples C1 and C2. In the case of C3, activity decreased, probably due to the absence of GA. In samples C4 and C5, Amicon^®^-filtered, a moderate increase in activity was observed.

Kinetic parameters were determined considering the specific activity of the conjugates (**Table 3**). The greatest improvement of specific activity was achieved for C5. For C1 and C2 samples, very moderate or no increase of specific activity was obtained. These parameters were not determined for C3, the non-GA control.

**Table 3:** Kinetic parameters and specific activity of conjugates.

Sample	Km (μM)	Vmax (μM/min)	Amount of CALB (ng)	Specific activity (μM.min^-1^.ng^-1^)	Fold
Commercial lipase	1822.56 ± 365.67	1232.74 ± 149.05	4.95E-05	2.49E+07	N/D
C1	2538.10 ± 364.72	211.43 ± 20.66	5.73E-06	3.69E+07	1.5
C2	2731.43 ± 989.31	226.36 ± 56.85	9.05E-06	2.50E+07	1.0
C4	2385.67 ± 689.15	926.70 ± 176.10	2.04E-05	4.53E+07	1.8
C5	859.54±197.75	829.10 ± 86.12	9.96E-06	8.32E+07	3.4

*Last column shows relative improvement in specific activity of conjugates, in comparison with free CALB*.

### Electron Microscopy

**FIGURE 3:**
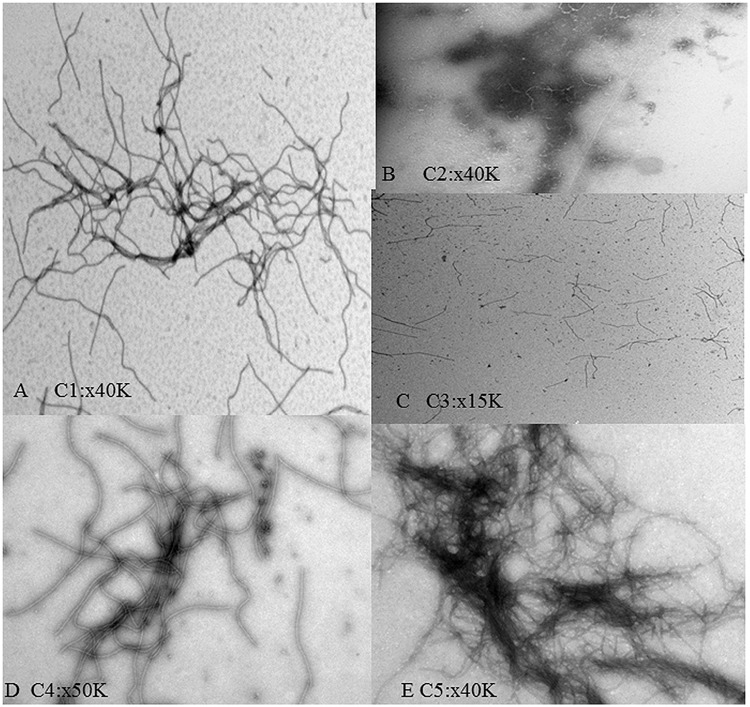
**Electron micrographs of the TuMV:CALB bioconjugates formed at different ratios. (A–E)** correspond to C1–C5 ratios, as described in **Table 1**. Magnifications used in each case are shown in the different panels.

Electron microscopy grids were prepared to confirm the expected presence of supramacromolecular structures in the conjugates. Micrographs corresponding to each are shown in **Figure 3**. In C1 a net was found showing a slight accumulation of dark material, most likely CALB, which seemed to form preferentially in knots of the nanonet. No viral particles were found in C2, and C3 showed a poor net formation. In C4 and C5, complex nets were observed, particularly in C5. C4 and C5 samples presented a heterogeneous distribution being more frequently found at the edges of the grids, an effect probably due to the increase of hydrophobicity in samples with high CALB content. The C5 nanonets tended to accumulate large amounts of the dark material in certain regions whereas other zones of the nets appeared as essentially CALB-free.

## Discussion

Chemical conjugation of CALB and TuMV virions using GA as a homobifunctional conjugating agent, allowed us to obtain TuMV-based nanonets on which large aggregates of CALB formed. Given the presence of amines on the surfaces of both CALB and the TuMV CP, the components of the complex nanonets are, presumably, covalently linked via imine formation mediated by the GA. These complex nanonets showed good CALB hydrolytic activity when tested using *p*-nitrophenyl acetate as a substrate, resulting in *p*-nitrophenol as a spectrophotometrically measurable product, and these showed an even higher specific activity compared with the commercially available free-enzyme. To the best of our knowledge, this is the first report of the formation of this type of nanonet involving protein subunits on the flexuous particles of TuMV. Remarkably these nanonets can be CALB-functionalized to provide catalytically active supramacromolecular complexes.

Enzyme nanoimmobilizations of plant-derived virions have been recently reported (Carette et al., 2007; Aljabali et al., 2012; Pille et al., 2013), extending from the initial concept of a ‘virus-based single enzyme nanoreactor’ (Comellas-Aragonès et al., 2007). In these earlier studies, both genetic and chemical virus-enzyme fusions were described, albeit with a limited number of enzyme molecules present in the supramacromolecular complexes. In our case, we cannot know how many enzyme molecules are retained in the virus-based nanonets, and it is not easy to assess the retention limit given the likely chemical nature of the retention. On the TuMV nets, CALB molecules seem to form structures with similarities to CLEAS (which we refer to as CLEAS-like), whose quantitative composition is not directly measurable. Nevertheless, like in CLEAS formed by enzymes alone, enzymatic activity is maintained and even increased. Nanonet structures cannot be formed based on icosahedral virions, rods being obvious basis for them. In our literature searches we have not found reports of functionalized nanonets using rigid virions such as, for example, tobacco mosaic virus particles. It is likely that flexuous rods, such as TuMV virions, are better-suited for the formation of three-dimensional nets. Electron micrographs show that these are indeed formed, and with a high degree of complexity.

We chose CALB to test the suitability of our system for nanoimmobilization, given its clear advantages. CALB is widely used as biocatalyst due to its many industrial applications in food, fuels, industrial chemistry and pharmacy, among others (Muñoz Solano et al., 2012). It has a good degree of regioselectivity and displays enantioselectivity toward secondary alcohols, amines, esters, and racemic acids (Anderson et al., 1998; Kirk et al., 2002; Gotor-Fernández et al., 2006; Juhl et al., 2010; Kołodziejska et al., 2012). In its immobilized form it is marketed under the name Novozym435, which is CALB immobilized on polymethylmethacrylate. It exhibits great stability against a wide range of pH values and temperature. Consequently it has been implemented for application in different organic reactions, including many used commercially (Kirk et al., 2002). In addition, CALB has been immobilized on many hydrophobic media with an adequate performance of immobilization, stability and recovery of enzyme activity (Fernández-Lorente et al., 2007). Enzyme immobilization on nanoparticles, or our present nanonet-based development, show similar advantages to their use on different supports since they allow easy removal of the reaction medium, reutilization of the biocatalyst, and increased stability – properties not easily achieved when free enzyme is used. Recently, CALB has been immobilized on different synthetic supports (for instance (Wu et al., 2013, 2015; Cipolatti et al., 2014; Valério et al., 2015). Our results demonstrate the value of an alternative approach.

For future industrial or semi-industrial applications, the nanonets must be able to withstand the physical and chemical stress arising during the enzyme conjugation process. Not much information is available with respect to the stability of plant flexuous rod virions in this regard, since most stability studies in the literature have paid special attention to infectivity, rather than to structural integrity. An easy and unsophisticated way to evaluate their potential structural changes is to take advantage of the A_250_/A_267_ ratio of structured nucleoproteins (Thouvenel et al., 1976). Thus, we subjected TuMV virions to different stress conditions such as pH range, exposure to different acetonitrile concentrations, and the combination time-temperature in the organic solvent mixture. The results of these tests (**Figure 2**) allowed us to verify that working conditions were in fact plausible for our system, and to set these conditions. Under appropriate conditions we were able to generate nanonets which incorporated the vast majority of the enzyme, provided that the ratio of enzyme-to-virion was large enough (**Tables 1** and **2**). Electron microscopy showed that the nets formed under these conditions displayed large protein aggregates, which seemed to build preferentially in knot regions, though this latter observation could not be clearly established (**Figure 3**). The complex nanonets displayed a slightly enhanced specific catalytic activity (**Table 3**). Enzyme aggregates that formed on the surface of these nanonets may be at an advantage compared to the immobilization achieved by genetic fusion to flexuous virus particles (Carette et al., 2007), or chemical fusion to icosahedral virions (Aljabali et al., 2012). The advantages of immobilizing enzymes in CLEAs have been recently reviewed (Sheldon and van Pelt, 2013). Several characteristics of the enzyme immobilization approach appear to be advantageous. For instance, it does not require a highly pure enzyme preparation (our commercial CALB was not a pure enzyme). Other important cited features include improved stabilization of the quaternary structures of multimeric enzymes, and better mass transfer properties. We think that these and other positive CLEA properties will be maintained when CLEA-like structures are formed on the virion-derived nanonets.

To our knowledge no previous approaches based on nanonets have been described for enzyme nanoimmobilization, and our work shows that this is a clear possibility. The resulting nanonets can be directly exploited to carry out biocatalytical processes, although it seems likely that the approach could be further improved, for instance by combining it with the use of carbon nanotubes (Zhao and Grüner, 2012). Applications in optoelectronics, energy generation and sensing are under study, and we are currently evaluating possible applications in biocatalysis.

## Author Contributions

SC, CM, and MA run the experiments. CY-C did the image analysis and quantification calculations. SC, CM, FS, JMS-M, and FP designed and discussed results of the experimental work. SC, FS, JMS-M, and FP wrote the script. FP conceived the original development.

## Conflict of Interest Statement

The authors declare that the research was conducted in the absence of any commercial or financial relationships that could be construed as a potential conflict of interest.

## Supplementary Material

FIGURE S1 **UV spectroscopy assessment of pH-induced structural changes in virion structure**.

FIGURE S2 **Bioconjugation of CALB and TuMV virions. (A)** Shows the chemical reactions involved. Symbols as in **Figure 1**. **(B)** Shows the gradient of color formation along the C1–C5 range of TuMV:CALB relative proportions.

FIGURE S3 **Estimation of the amount of virion-conjugated CALB by image analysis-1.** The **(upper)** shows an SDS-PAGE in which the Coomasie Blue staining has been converted to gray for the subsequent image analysis. Lane explanation is given in the **(lower)**, together with the parameter values obtained in the image quantitative analysis. Briefly, Mean Gray Values (MGV) was obtained in Adobe Photoshop for the two CALB zones (monomer and dimer) in the gel, and the differences with the background were calculated. These figures were then used for the subsequent calculations shown in **Supplementary Figure S4**.

FIGURE S4 **Estimation of the amount of virion-conjugated CALB by image analysis-2. (Left)** Shows a graphic representation of the values obtained in **Supplementary Figure S3**. **(Upper right)** Shows the regression analysis. **(Lower right)** Shows the quantification obtained.

FIGURE S5 **Kinetic curves of the different conjugates.** Velocity vs. substrate concentration is represented. Amounts of CALB used per assay are shown.
