# Supplementary figures and images for: Nanonets Derived from Turnip Mosaic Virus as Scaffolds for Increased Enzymatic Activity of Immobilized Candida antarctica Lipase B
Source: Front Plant Sci. 2016 Apr 11;7:464. doi: 10.3389/fpls.2016.00464 (PMC4826883; doi:10.3389/fpls.2016.00464)

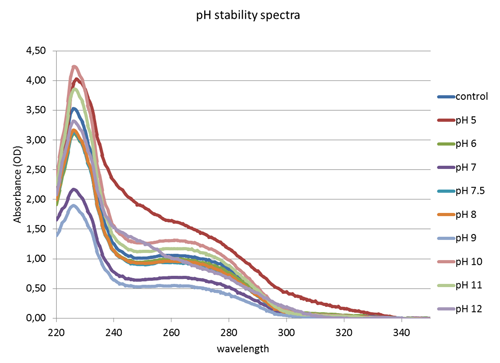

Supplement: FIGURE S1 — UV spectroscopy assessment of pH-induced structural changes in virion structure. [file Image_1.TIF]

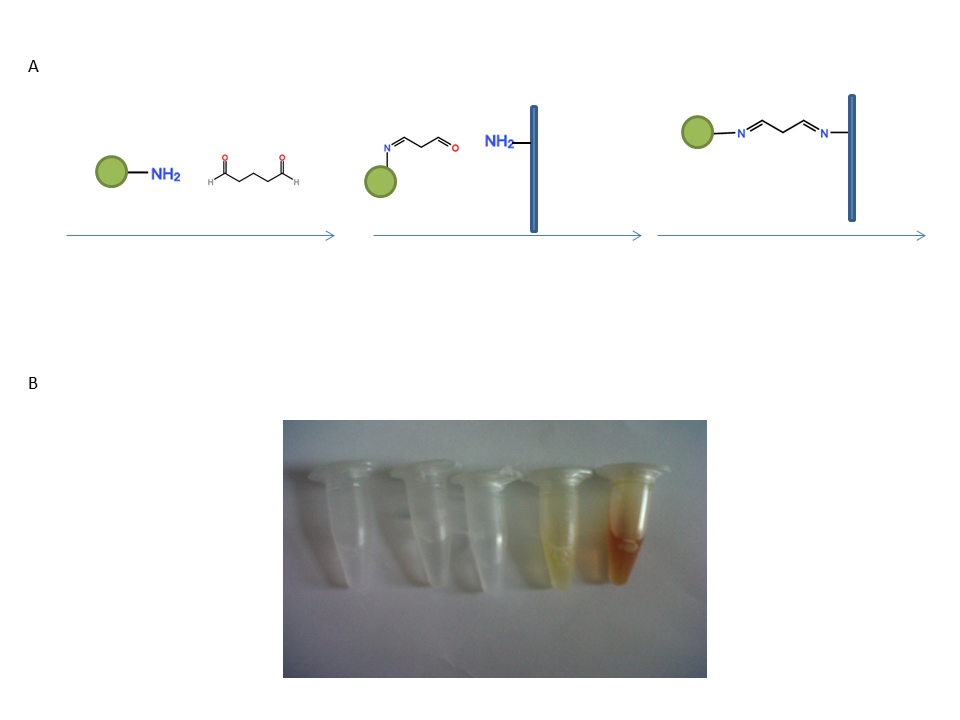

Supplement: FIGURE S2 — Bioconjugation of CALB and TuMV virions. (A) Shows the chemical reactions involved. Symbols as in Figure 1. (B) Shows the gradient of color formation along the C1–C5 range of TuMV:CALB relative proportions. [file Image_2.TIF]

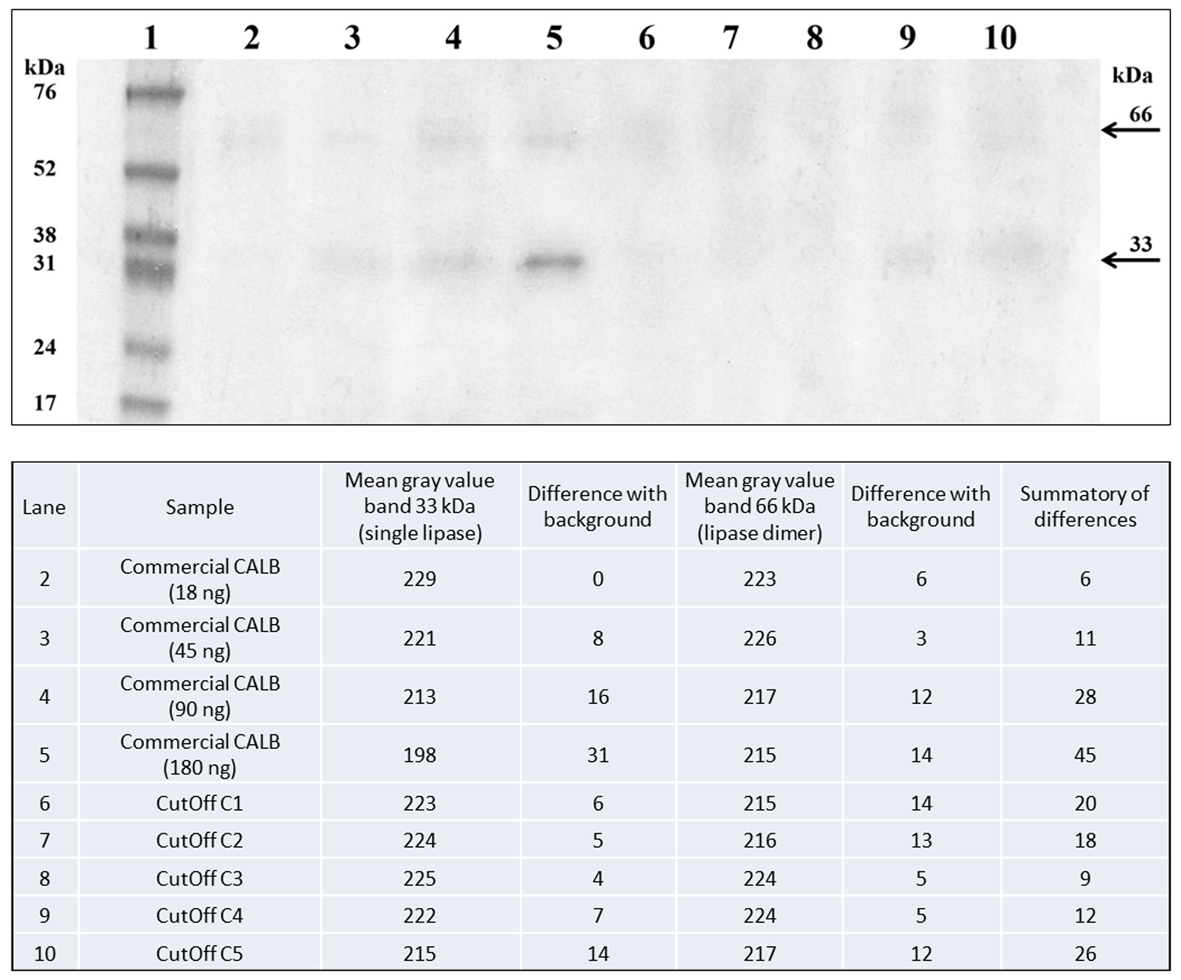

Supplement: FIGURE S3 — Estimation of the amount of virion-conjugated CALB by image analysis-1. The (upper) shows an SDS-PAGE in which the Coomasie Blue staining has been converted to gray for the subsequent image analysis. Lane explanation is given in the (lower), together with the parameter values obtained in the image quantitative analysis. Briefly, Mean Gray Values (MGV) was obtained in Adobe Photoshop for the two CALB zones (monomer and dimer) in the gel, and the differences with the background were calculated. These figures were then used for the subsequent calculations shown in Supplementary Figure S4. [file Image_3.TIF]

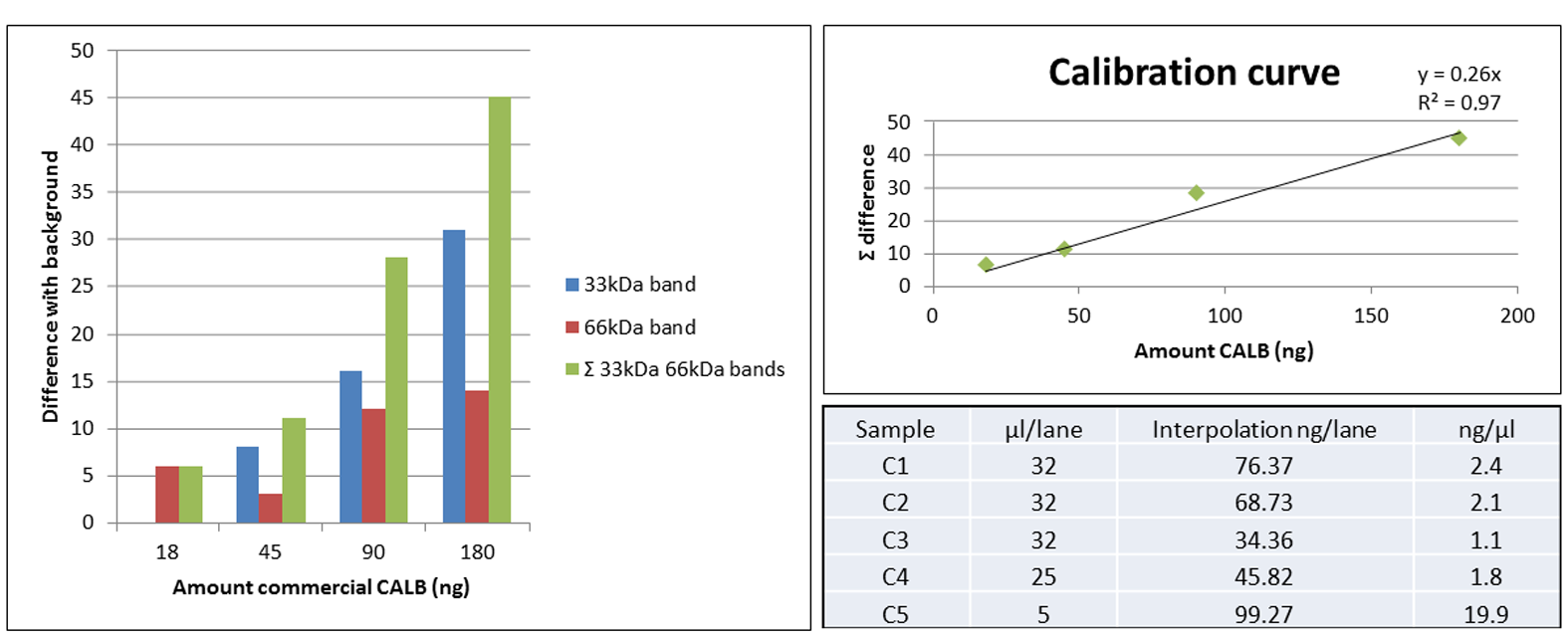

Supplement: FIGURE S4 — Estimation of the amount of virion-conjugated CALB by image analysis-2. (Left) Shows a graphic representation of the values obtained in Supplementary Figure S3. (Upper right) Shows the regression analysis. (Lower right) Shows the quantification obtained. [file Image_4.TIF]

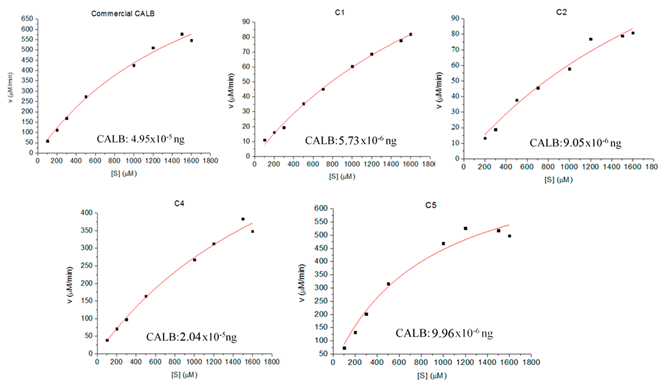

Supplement: FIGURE S5 — Kinetic curves of the different conjugates. Velocity vs. substrate concentration is represented. Amounts of CALB used per assay are shown. [file Image_5.TIF]
